# A novel melanoma prognostic model based on the ferroptosis-related long non-coding RNA

**DOI:** 10.3389/fonc.2022.929960

**Published:** 2022-10-12

**Authors:** Yamin Rao, Jinchao Zhu, Haiyan Zheng, Wei Dong, Qingyuan Lin

**Affiliations:** ^1^ Department of Pathology, Shanghai Ninth People’s Hospital, Shanghai Jiao Tong University School of Medicine, Shanghai, China; ^2^ Department of Pathology, Eastern Hepatobilliary Surgery Hospital, The Second Military Medical University, Shanghai, China

**Keywords:** ferroptosis, melanoma, prognosis model, lncRNA, bioinformatics analysis

## Abstract

Ferroptosis is an iron-dependent programmed cell death related to the biological process of many kinds of tumors. Long noncoding RNAs (*LncRNA)* have been found to play essential roles in the tumor, and their functions in the ferroptosis of tumor cells have been partially discovered. However, there is no summary of ferroptosis-related *LncRNA* and its functions in melanoma. In the present study, we aim to explore the expression profile of ferroptosis-related *LncRNA* genes and their value in melanoma prognosis by bioinformatics analysis. The expression of ferroptosis-related gene (*FRG*) from melanoma clinical data was extracted based on the Cancer Genome Atlas (*TCGA*) database. By screening the *RNA* expression data of 472 cases of melanoma and 810 cases of normal skin, eighteen ferroptosis-related differential genes were found to be related to the overall survival rate. Furthermore, 384 ferroptosis-related *LncRNAs* were discovered through constructing the *mRNA-LncRNA* co-expression network, and ten of them were found with prognostic significance in melanoma by multivariate Cox analysis. Risk assessment showed that the high expression of *LncRNA00520* is associated with poor prognosis, while the increased expression of the other LncRNA is beneficial to the prognosis of patients with melanoma. From univariate and multivariate Cox regression analysis, there were ten ferroptosis-related LncRNA risk models towards to be significant independent prognostic factors for patients with melanoma and valuable predictive factors for overall survival (*OS*)(P<0.05). The *ROC* curve further suggested that the risk score has relatively reliable predictive ability (*AUC*=0.718). The protein level of ferroptosis-related genes was verified by the *HPA* database and *IHC* test, leading to the discovery that the expressions of *ALOX5*, *PEBP1*, *ACSL4*, and *ZEB1* proteins up-regulated in tumor tissues, and existed differences between tumor tissues and normal tissues. In conclusion, we identified ten ferroptosis-related *LncRNA* and constructed a prognosis model base.

## Introduction

Although melanoma is not the most common malignant tumor in skin cancer, it is the leading cause of skin cancer-related death (1, 2), The occurrence and development of melanoma are related to various complex factors, including lifestyle, living environment, genetic and epigenetic changes. Melanoma has significant heterogeneity which would be a major determinant of invasion and metastasis, and its occurrence is closely related to the aberrant regulation of many genes and signal pathways. Increasing evidences showed that many genes involved the development and progression of melanoma, most recurrent mutation is BRAFV600E, which hyperactivates the mitogen-activated protein kinase (MAPK) pathway (3) followed by NRAS mutation (4) and NF1 mutations (5). Among the regulatory pathways implicated in melanoma, MAPK pathway is most common, others include PI3K pathway, Wingless type MMTV integration site (Wnt) signaling (6). Although the treatment of melanoma has been greatly improved in recent years, the prognosis of tumor patients remains poor due to individual differences. The effect of standard chemotherapy on patients with progressive disease is mainly limited, with only 5-10% effective rate (7) and a 27% 5-year survival rate of metastatic melanoma (8). The acquisition of melanoma treatment strategies depends on an accurate understanding of the exact mechanism of the occurrence and development of melanoma. The search for sensitive and accurate biomarkers will better predict the survival and prognosis of patients with melanoma and improve the therapeutic effect.

As well known, cancer cells, including melanoma cells, have an anti-apoptotic property and exhibit malignancy. Ferroptosis is an iron-dependent cell death dependent on the intracellular accumulation of reactive oxygen species (ROS), distinct from apoptosis and autophagy (9). As a non-apoptotic modality of cell death, ferroptosis, is defined as an iron-dependent regulated necrosis that is caused by massive lipid peroxidation-mediated membrane damage featured by a necrosis-like morphological change (10). It is a ROS-dependent form of cell death associated with iron accumulation and lipid peroxidation, involving a series of genes and protein expression including Acyl-CoA synthetase long-chain family member 4 (ACSL4) (11, 12). It determines cell fates through immune and inflammatory reactions. As a form of inflammatory cell death, ferroptosis can release lipid oxidation products to activate NF-κB pathway during tissue injury or tumor therapy.

Increasing evidence indicated that ferroptosis plays an essential role in cancer (12). Dysregulation of iron metabolism is a risk factor for cancer and can promote tumor growth. Therefore, understanding the mechanism of ferroptosis in cancer is of great significance for developing new methods of diagnosis and treatment. Furthermore, long non-coding RNA (Long non-coding RNAs, lncRNAs) is a subset of RNA molecule with more than 200 nucleotidess and lack of protein-coding ability. lncRNAs interact with mRNAs, DNA, proteins and microRNAs (miRNAs) and modulating epigenetic, transcriptional, post-transcriptional, translational and post-translational events of gene expression in mutlitlevels (13). with four interactional ways such as signal, decoy, guide and scaffold (14). LncRNA performs its functions mainly through lncRNA-protein interaction, lncRNA-ceRNA network, lncRNA-miRNA-mRNA silencing, binding to DNA in cis and binding to promoter regions of encoding genes (15). LncRNA participates in the occurrence and development of many kinds of tumors (16–19). Cancer therapy such as chemotherapy could be modulated by LncRNAs (20). so LncRNAs can be used as diagnostic, therapeutic and prognostic markers in cancers (21, 22).

Although the study of ferroptosis is a relatively new field with fast development, it is still a lack of studies on ferroptosis-related LncRNA genes in melanoma. Thus, this study is aimed to explore the expression profile of ferroptosis-related LncRNA and their value in the prognosis of melanoma by bioinformatics analysis. Meanwhile, using the HPA protein database, we probed into the proteins linked to ferroptosis-related LncRNA genes.

## Methods

### Data collection

RNA sequence (tumor) data and corresponding clinical information of 1416 skin melanoma patients was extracted from TCGA database. The gene expression profiles were normalized by “Limma” R software installation package. The gene expression of normal skin tissue was extracted from the GTEX data sets from UCXC database. Then the intersection of the genes of TCGA and GTEX data sets was made and merged. Both TCGA data and UCXC data are public, thus the study exempts the approval of local ethics committees.

### Identification of ferroptosis-related genes

The differentially expressed genes in tumor and non-tumor tissues in the TCGA cohort were screened by “LIMMA” R software package. The overall survival rate (OS) was analyzed by univariate Cox analysis to screen ferroptosis-related genes with prognostic value. Subsequently, heat map visualization, PPI (Protein-Protein Interaction Networks, https://cn.string-db.org/) network interaction and co-expression correlation analysis on these genes were performed.

### Construction and verification of ferroptosis-related lncRNAs

We constructed ferroptosis-related mRNA-lncRNA co-expression network *via* using ferroptosis-related genes with prognostic value according to the correlation coefficient > 0.3 and P < 0.01. Then we identified 384 ferroptosis-related lncRNA using “LIMMA” R software package for Pearson correlation analysis.

### Development of ferroptosis-related lncRNA prognostic signatures

We conducted a univariate Cox proportional hazard analysis according to the criteria of P < 0.01. Then, we carried out multivariate Cox analysis using “survival” R package to establish the optimal prognostic risk model based on the risk score (coef (lncRNA1) × expr(lncRNA1) + coef(lncRNA2) × expr(lncRNA2) +… + coef (lncRNAn) × expr(lncRNAn). Coef (LncRNAn) is defined as the lncRNAs coefficient related to survival. ExpR (LncRNAn) is defined as the expression of lncRNAs. According to the median risk score, melanoma patients in TCGA were divided into high-risk group and low-risk group. Kaplan-Meier survival analysis used survival and Servicer R package to estimate the survival difference between the two groups.

### Independent prognostic analysis and ROC curve drawing

In order to evaluate the relationship between survival and clinicopathological factors and risk score, univariate and multivariate Cox regression analysis was performed using the SurvivalR software package. The time-dependent receiver operating characteristic (ROC) curve was drawn by the Survival ROC R software package, and the prediction accuracy of different clinicopathological factors and risk scores on survival time was estimated.

### Gene enrichment analysis

Gene set enrichment analysis (GSEA) was used for functional annotation. GSEA (https://www.gSea-msigdb.Org/gsia/index.jsp) is a powerful analytical method to explain the whole genome expression profile, and detect the relationship between several cancer-related pathways, metabolic pathways, transcriptional procedures and stress responses in biological processes. P < 0.05 was considered to be statistically significant.

### HPA protein database

HPA (human protein atlas) protein database (https://www.proteinatlas.org/) uses transcriptome and proteomics techniques to study protein expression in different human tissues and organs at RNA and protein levels. Up to now, more than 26000 kinds of antibodies have been collected in HPA. The protein expression information in HPA was from the immunohistochemistry (IHC) staining and confirmed by professionals with high accuracy and credibility. Therefore, we use the HPA database to verify the protein level of ferroptosis-related genes.

### Immunohistochemistry analysis of clinical samples

Immunohistochemical studies were performed on formalin-fixed paraffin-embedded (FFPE) tissues, and used monoclonal antibodies including anti-ALOX5 (EP6072, dilution 1:100, Abcam), Anti-PEBP1 (EPR2875Y, dilution 1:250, Abcam), Anti-ACSL4 (EPR8640, dilution 1:500, Abcam) and Anti-ZEB1 (EPR17375, dilution 1:1000, Abcam), which was operated on BENCHMARK automatic immunohistochemical instrument. Positive and negative controls were carried out according to the manufacturers’ recommendations. Immunoreactivity result was recorded as negative without tumor cell expression, conversely as positive.

### Statistical analysis

All statistical analyses were carried out using R software (version 4.0.3). Normally and non-normally distributed variables were analyzed using the unpaired student’s t-test and the Wilcoxon test, respectively. The co-expression networks of ferroptosis-related lncRNAs-mRNAs with prognostic value were established. Based on FDR, Benjamini-Hochberg method was used to identify the differently expressed lncRNAs.

## Results

### Identification of prognostic factors related to ferroptosis in TCGA cohort

**Figure 1:**
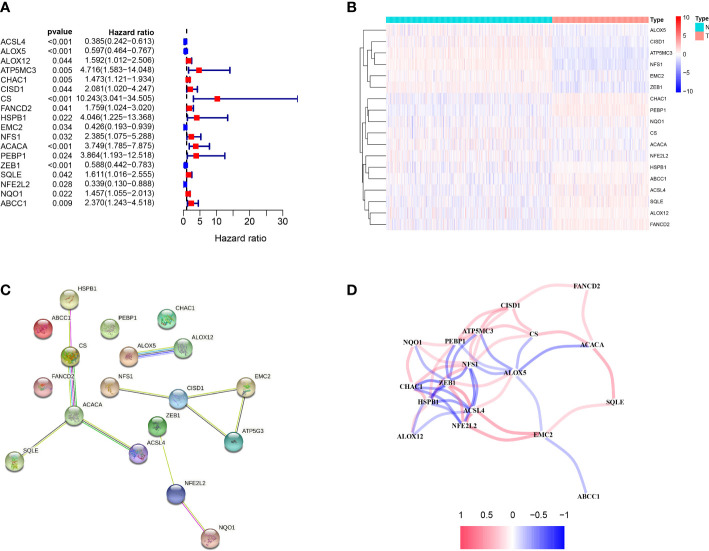
Identification of candidate ferroptosis-related genes in TCGA cohort. **(A)** Forest plot of univariate Cox regression analysis. **(B)** The expression of 18 genes in cancer tissues. **(C)** The interaction between 18 candidate genes in the PPI network. **(D)** The correlation network of candidate genes. The correlation coefficients are represented by different colors.

We uncovered the differentially expressed ferroptosis-related genes in tumor tissues and paracancerous tissues. Univariate Cox regression analysis identified 18 OS-associated genes (**Figure 1A**). The genes were clustered to generate a heat map,and the map showed CHAC1, P-EBP1, NQO1, HSPB1, ABCC1, ACSL4, SQLE, ALOX12, FANCD2 are highly expressed, and they are related to the poor prognosis of melanoma patients. In contrast, ALOX5, CISD1, ATP5MC3, NFS1, EMC2, ZEB1 are highly expressed in normal tissues, and they are related to the good prognosis of melanoma patients (**Figure 1B**). Protein interaction network analysis showed that ACACA was a central gene (**Figure 1C**), and the correlation between genes was shown in **Figure 1D**.

### Identification of ferroptosis-related lncRNA with important prognostic value In melanoma

**Figure 2:**
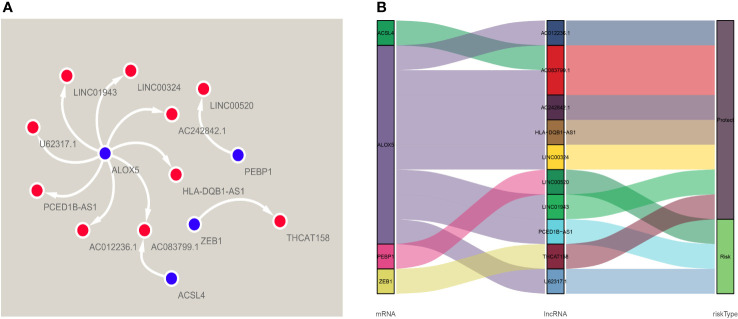
A co-expression network of four ferroptosis-related-lncRNAs-mRNAs with predictive value. **(A)** The co-expression relationship between ferroptosis genes and lncRNA, visualized by Cytoscape. **(B)** The co-expression of iron death gene and lncRNA and the correlation of risk value, visualized by Sankey diagram.

A total of 384 ferroptosis-related LncRNAs were obtained by constructing a co-expression network of 18 ferroptosis-related encoding genes (mRNA) with prognostic value. COX regression analysis showed that 46 ferroptosis-related LncRNAs were significantly associated with TCGA melanoma patients (P <0.01), 37 of which were low risk (risk ratio: HR <1), 9 as high-risk (risk ratio: HR> 1). Multivariate Cox analysis further screened 10 prognostic ferroptosis-related LncRNAs. These 10 LncRNAs form an optimal pre-rear risk model of ferroptosis-related LncRNA. As shown in **Figures 2A, B**, we also established 10 visualized ferroptosis-related LncRNAs-mRNA co-expression networks with prognostic value.

**Figure 3:**
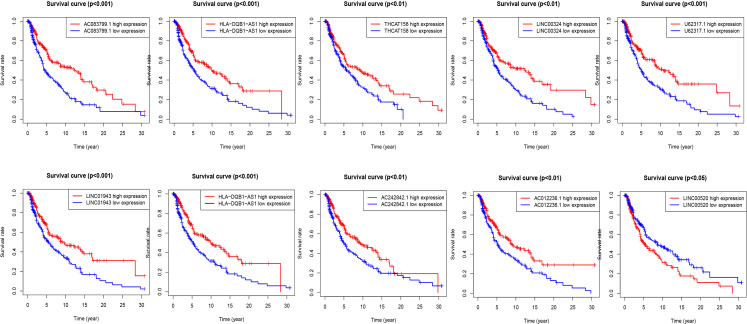
Kaplan-Meier survival curves of lncRNAs that predict the prognosis of melanoma in the TCGA data set. Ten ferroptosis-related-LncRNA were found to be independent prognostic factors for melanoma patients. Nine of them (THCAT158, AC083799.1, AC012236.1, LINC01943, HLA-DQB1-AS1, LINC00324, AC242842.1, PCED1B-AS1, U62317.1) are favorable prognostic factors for melanoma patients, and one of them (LINC00520) is an unfavorable prognostic factor for melanoma.

According to the risk score formula and the calculated median risk score, we divided melanoma patients into high-risk and low-risk groups. In this study, we drew ten lncRNA survival curves with prognostic value. Despite that LncRNA00520 represents the poor prognosis of patients with melanoma, the high expression of the other several LncRNAs was beneficial to the prognosis of patients with melanoma (**Figure 3**).

**Figure 4:**
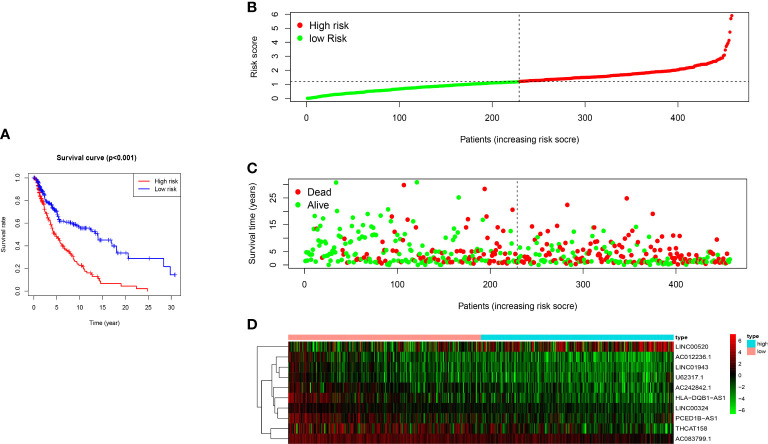
The prognostic value of 10 ferroptosis-related-lncRNA risk models in the TCGA cohort. **(A)** Kaplan-Meier survival analysis of high-risk and low-risk populations based on risk model and median risk score. **(B)** Risk curve based on the risk score of each sample. **(C)** Scatter plot based on the survival status of each sample. The green dot and the red dot represent survival and death, respectively. **(D)** The heat map shows the expression levels of ferroptosis-related-LncRNA in the high-risk and low-risk groups.

Kaplan-Meier survival analysis demonstrated that the overall survival rate (OS) of the high-risk group was worse than that in the low-risk group (p <0.001) (**Figure 4A**). The risk score had a specific predictive value for patients’ prognoses. We use a risk curve and scatter plot to describe the risk score and related survival status of patients with melanoma. The results showed that mortality depended on the risk score (**Figures 4B, C**). The heat map showed that the expression of LINC00520 was highly expressed in the high-risk group, while the expression of AC012236.1, LINC01943, U62317.1, AC242842.1, HLA−DQB1−AS1, LINC00324, PCED1B−AS1, THCAT158, AC083799.1 was separately up-regulated in the low-risk group (**Figure 4D**). Therefore, the above studies identified ten LncRNAs associated with ferroptosis, which is significant for the melanoma prognosis.

### Evaluation of risk model using 10 ferroptosis-related lncRNA as independent prognostic factors in patients with melanoma

**Figure 5:**
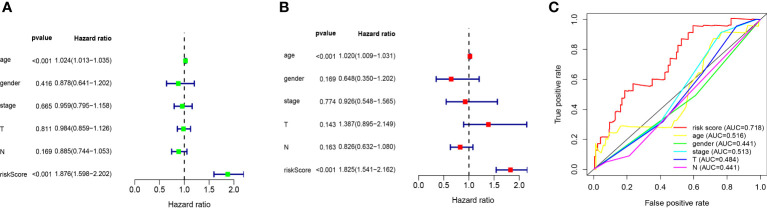
Evaluation of ten ferroptosis-related-lncRNA prognostic risk models in melanoma. **(A)** Univariate and **(B)**, multivariate Cox regression analysis of risk model score and clinical characteristics on prognosis. **(C)** The risk model score and AUC of clinical characteristics were calculated according to the ROC curve. Clinical features include age, sex, stage and TNM stage.

We performed univariate and multivariate Cox regression analysis to evaluate whether the above ten ferroptosis-related LncRNA risk models are independent prognostic factors for patients with melanoma. In univariate COX regression analysis (**Figure 5A**), the risk score and 95%CI (HR) were 1.876 and 1.598-2.202 (P < 0.001), respectively, and in multivariate COX regression analysis (**Figure 5B**), they were 1.825 and 1.541-2.162 (P < 0.001), respectively. It is suggested that the risk models of 10 ferroptosis-related lncRNA are the most significant prognostic factors for melanoma. In order to evaluate the sensitivity and specificity of risk score in predicting the prognosis of melanoma patients, the area under the ROC curve (AUC) of the risk score was calculated (**Figure 5C**). The AUC value of the risk score was 0.718, indicating that the prognosis risk model of 10 melanoma iron death related lnc RNAs was quite reliable. Therefore, 10 risk models of iron death related lncRNA are important independent prognostic factors for melanoma cancer patients.

### GSEA enrichment analysis

**Figure 6:**
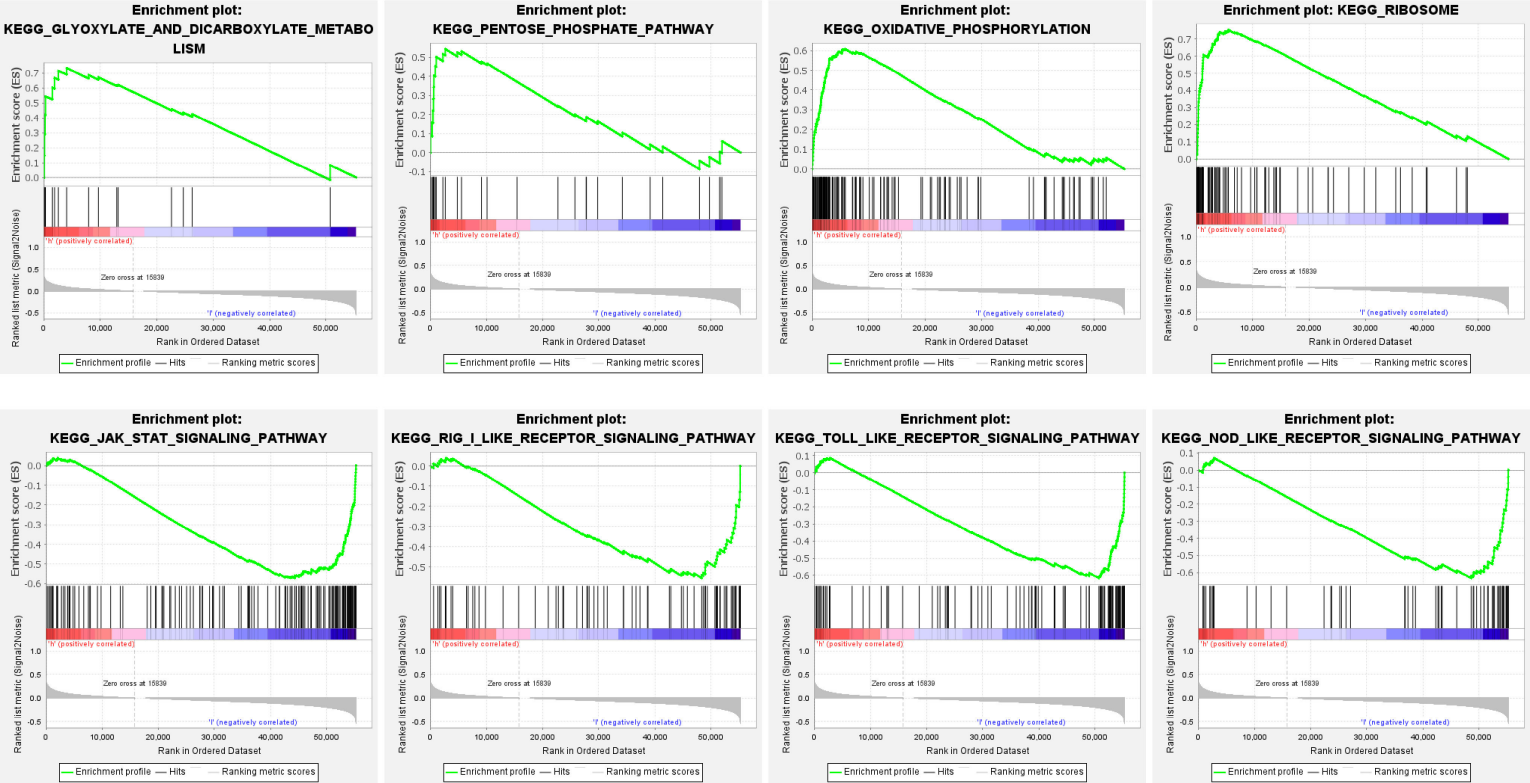
Functional enrichment analysis of ten ferroptosis-related-lncRNA risk models based on GSEA.

Functional pathway enrichment is based on Gene Set Enrichment Analysis (GSEA). The results revealed that most of the ten ferroptosis-related lincRNAs correlate with the metabolism signaling pathway. In the high expression group, ferroptosis-related lincRNAs regulated the glyoxylate and dicarboxylate metabolism signaling pathway, oxidative phosphorylation signaling pathway, and pentose phosphate pathway. As meanwhile, in the low expression group, they trend to affect the JAK-STAT signaling pathway, nod like receptor signaling_pathway, and toll-like receptor signaling pathway (**Figure 6**, **Table 1**).

**Table 1:** Gene enrichment analysis.

MSigDB collection	Gene set name	NES	FDR q-value	p-value
c2.cp.kegg.v7.2.symbols.gmt	KEGG_GLYOXYLATE_AND_DICARBOXYLATE_METABOLISM	1.95	0.07	0.00
[Curated]	KEGG_JAK_STAT_SIGNALING_PATHWAY	-2.04	0.004	0.00
	KEGG_NOD_LIKE_RECEPTOR_SIGNALING_PATHWAY	-2.02	0.005	0.00
	KEGG_OXIDATIVE_PHOSPHORYLATION	1.75	0.30	0.02
	KEGG_PENTOSE_PHOSPHATE_PATHWAY	1.62	0.40	0.03
	KEGG_RIBOSOME	1.69	0.32	0.02
	KEGG_RIG_I_LIKE_RECEPTOR_SIGNALING_PATHWAY KEGG_TOLL_LIKE_RECEPTOR_SIGNALING_PATHWAY	-1.88 -2.09	0.016 0.001	0.00 0.00

### HPA and immunohistochemistry (IHC) verification

**Figure 7:**
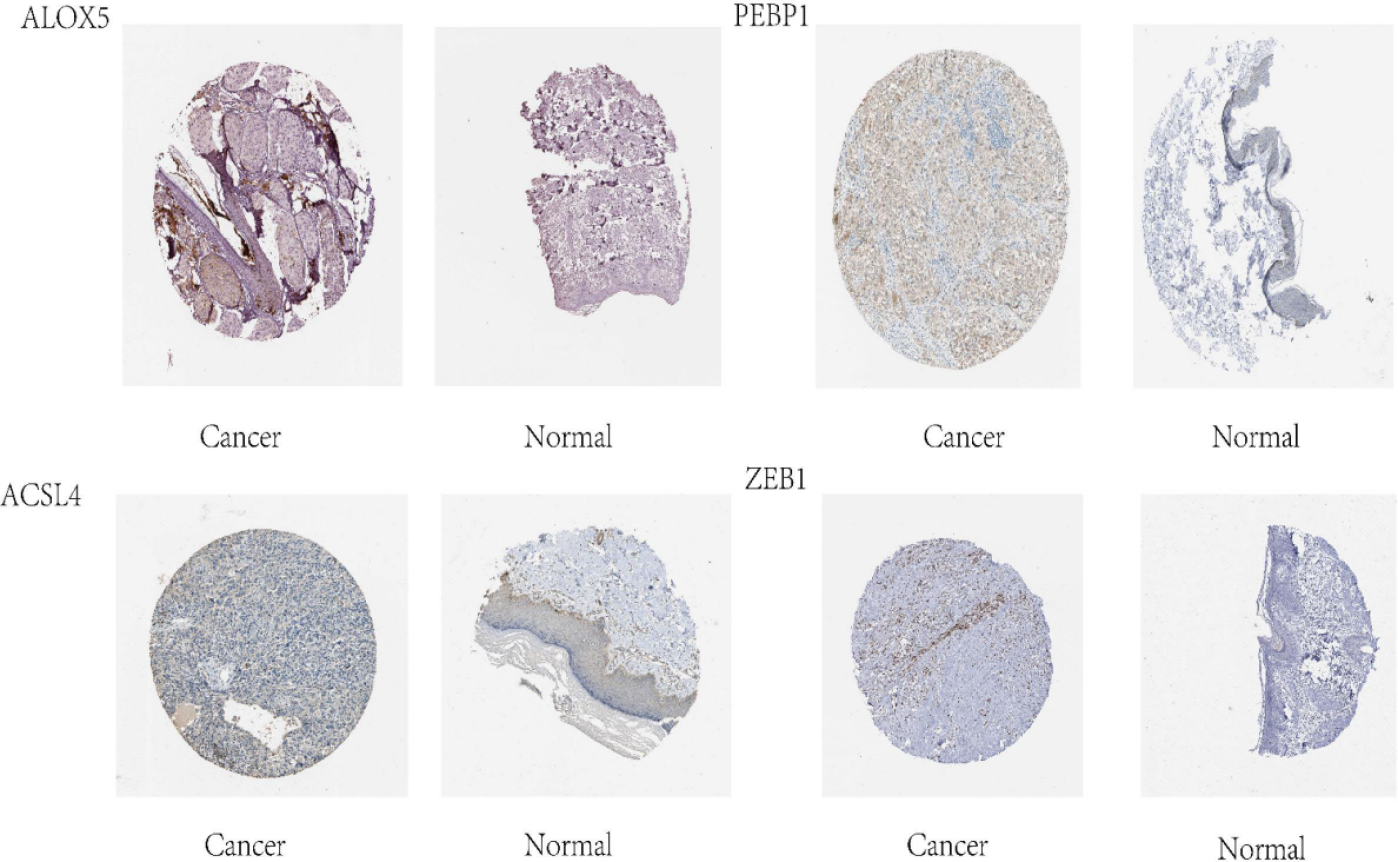
Four ferroptosis genes in melanoma identified in the HPA database.

**Figure 8:**
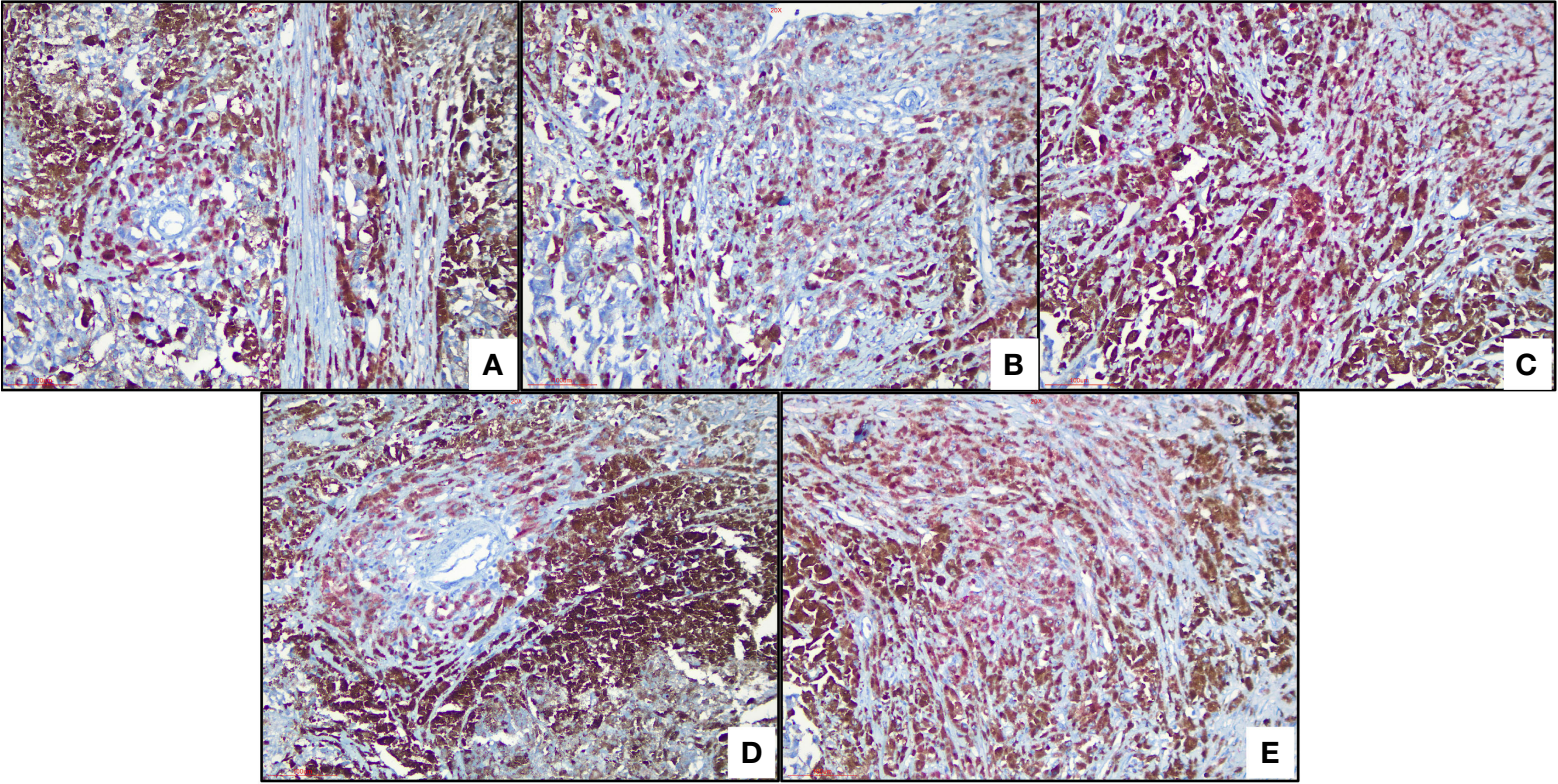
Immunohistochemical analysis of ferroptosis-related proteins **(A)** Cytoplastic expression of ALOX5 (red color means positive) **(B)** Cytoplastic expression of PEBP1 (red color means positive). **(C)** Expression of ACSL4, (red color means positive) **(D)** ZEB1 (red) and **(E)** ZEB1 (red) stain the cytoplasm of tumor cells.

We used the human protein map (http://www.proteinatlas.org) database to verify four ferroptosis-related genes. The results showed that all four ferroptosis-related genes were expressed in varying degrees in melanoma, as shown in **Figure 7**. We observed expressions of ferroptosis related proteins including ALOX5, PEBP1, ACSL4 and ZEB1 in melanoma, which showed a strong cytoplasmic staining (**Figure 8**).

## Discussion

In recent years, the etiology and pathogenesis of melanoma are constantly being studied to expand its therapeutic areas. Ferroptosis is a newly discovered form of cell death that is different from apoptosis, typical necrosis and autophagy (9). The ferroptosis does not depend on the caspase activity and receptor-interacting protein 1 (RIPK1) kinase activity, but depends on the iron-dependent accumulation of lipid hydrogen peroxide to a lethal level (10). The increasing evidence indicated that ferroptosis plays a vital role in many kinds of tumor progression and prognosis (23–25). including melanoma. Luo et al. (26) reported the ferroptosis-suppressing effect of miR-137. However, many critical issues such as the interconnection between ferroptosis and other regulators of gene expression remain unsolved. This study successfully obtained the expression profile of ferroptosis-related genes in melanoma and identified eighteen ferroptosis-related genes with prognostic value by survival analysis. Through the analysis of mRNA LncRNA co-expression network, we constructed novel, well-performing risk prediction models based on the 10 LncRNA for melanoma patients.

As well known, LncRNA affects the target gene expression through epigenetic modification and transcriptional regulation. It is reported that LncRNA is abnormally expressed and related to the malignant progression of melanoma (27, 28). LncRNA-mRNA co-expression analysis is a standard method for identifying potential target genes of lncRNA and exploring the biological function of lncRNA in diseases. In this study, based on the ferroptosis-related mRNA-lncRNA co-expression network, we identified 384 ferroptosis-related lncRNA and obtained an optimal risk model including 10 LncRNAs (THCAT158, AC083799.1, AC012236.1, LINC01943, HLA-DQB1-AS1, LINC00324, AC242842.1, LINC00520, PCED1B-AS1, U62317.1). Furthermore, we found LINC00520 is an adverse prognostic factor for melanoma by multivariate Cox analysis, LINC00520 was reported to play roles in the occurrence and development of a variety of solid tumors, such as breast cancer (29, 30). However, LINC00520 is a new oncogene in melanoma, of which the exact role and molecular mechanism in malignant melanoma remain unclear. Mei XL et al (31) has found that LINC00520 inhibits the growth and metastasis of skin squamous cell carcinoma. Luan W et al. found that LICN00520 is highly expressed and promotes melanoma cells’ the proliferation, invasion, and migration. It is a risk factor for the prognosis of melanoma patients and might be used as a survival biomarker or therapeutic target for melanoma (32). In this study, survival analysis revealed that LINC00520 was highly expressed in melanoma in the high-risk group, suggesting that the prognosis of this group was poor, but the conclusion still needs to be further verified.

GO enrichment analysis revealed that the ferroptosis-related LncRNA enrichment regions differed between the high expression and the low expression groups. In the high expression group, ferroptosis-related LncRNA is closely related to glyoxylate and dicarboxylate metabolism, oxidative phosphorylation and so on. While the low expression group mainly focused on pathways such as JAK-STAT signaling, NOD-like receptor signaling pathway, RIG-I-like receptor signaling pathway and Toll-like receptor signaling.

In addition, ten lncRNA were analyzed with the human protein database HPA (http://www.proteinatlas.org), and four ferroptosis-related proteins were obtained to understand further the protein function ALOX5, PEBP1, ACSL4 and ZEB1. Moreover, IHC verification in subsequent clinical specimens showed that these ferroptosis-related proteins were highly expressed in melanoma compared with normal tissues. ALOX5, belonging to the Human lipoxygenases (LOX) family (33), is a newly discovered ELF3 target gene, and related to host immune response. It has been proved that ALOX5 can stimulate the expression of oncogenes and promote mitosis and chemotaxis in various types of cancer cells (34–36). It may also promote tumorigenesis, angiogenesis and tumor cell invasion by regulating tumor cell oxidation, arachidonic acid metabolism, carcinogenic signaling pathway and inflammation (37). Although a previous study has shown an association between ALOX5 with poor outcome of tumor (36), there are no studies indicating association of ALOX5 with the prognosis of melanoma, we herein show for the first time that ALOX5 is associated with the prognosis of melanoma.

Moreover, phosphatidylethanolamine binding protein 1 (PEBP1) is a conserved protein, isolated initially based on its ability to bind phospholipids (38). PEBP1 acts as an endogenous inhibitor of the RAF-MEK-ERK pathway by directly blocking the activation of RAF-1 proto-oncogene serine/threonine protein kinase (RAF1) (39). This concurrent activation of autophagy protects cells from ferroptosis death and the release of mitochondrial DNA. The mechanism through which LncRNA regulates PEBP1 is unknown. Our results suggest that PEBP1 may affect the prognosis of melanoma by LncRNA.

Acyl-CoA synthetase long-chain family member 4 (ACSL4) is a vital isoenzyme in the metabolism of polyunsaturated fatty acids(polyunsaturated fatty acids, PUFA), which determines the sensitivity of ferroptosis. ACSL4 disorders are associated with a variety of malignant tumors, including gastric (40), breast (41) and liver cancer (42, 43). Previous studies showed ACSL4 displayed both tumor-promoting and tumor-suppressive functions in different tumor types, while the role of ACSL4 in melanoma was still elusive. Our results provides new insights of ACSL4 into the mechanism of melanoma prognosis and provide a theoretical basis for developing new therapeutic strategies and drugs in the future.

Zinc-fingerE-box-bindinghomeobox1 (ZEB1) is a transcriptional regulator of epithelial-mesenchymal transformation of (EMT) (44, 45). Consisting of two zinc finger clusters and a central homologous domain that binds to DNA (46). The study of Li L shows that ZEB1 is overexpressed in hepatocellular carcinoma, which promotes tumor cell migration, invasion, and metastasis through EMT (47). Besides, ZEB1 reverses the more primitive state of melanoma (a neural crest stem cell (NCSC)) into a more differentiated state and reduces drug resistance to related treatments (48). Our results revealed the correlation between the ZEB1 expression and melanoma prognosis and suggested that targeting ZEB1 might be a potential method for treating melanoma.

## Conclusion

In the present study, we systematically employed bioinformatics to analyze the ferroptosis-related differential genes in melanoma and identified independent prognostic differential genes. Through the association of ferroptosis-related mRNA-LncRNA, an effective prognostic model based on the risk score was successfully constructed. Our results provided a direction for prognosis assessment in melanoma patients, and the four ferroptosis-related LncRNA might be used as new biomarkers for melanoma.

## Data availability statement

The original contributions presented in the study are included in the article/supplementary material. Further inquiries can be directed to the corresponding author.

## Author contributions

YR and JZ carried out the studies, participated in collecting data, and drafted the manuscript. HZ and WD performed the statistical analysis and participated in its design. QL provided help and advice on administration. YR, JZ, HZ, and QL wrote the manuscript. All authors contributed to editorial changes in the manuscript. All authors read and approved the final manuscript.

## Funding

This work was supported by the Project of biobank (No.YBKB201911) from Shanghai Ninth People’s Hospital, Shanghai Jiao Tong University School of Medicine and the Cross disciplinary Research Projects of Ninth People’s Hospital, Shanghai JiaoTong university School of Medicine (JYJC202106).

## Conflict of interest

The authors declare that the research was conducted in the absence of any commercial or financial relationships that could be construed as a potential conflict of interest.

## Publisher’s Note

All claims expressed in this article are solely those of the authors and do not necessarily represent those of their affiliated organizations, or those of the publisher, the editors and the reviewers. Any product that may be evaluated in this article, or claim that may be made by its manufacturer, is not guaranteed or endorsed by the publisher.
